# Virtual Sensors for Advanced Controllers in Rehabilitation Robotics

**DOI:** 10.3390/s18030785

**Published:** 2018-03-05

**Authors:** Aitziber Mancisidor, Asier Zubizarreta, Itziar Cabanes, Eva Portillo, Je Hyung Jung

**Affiliations:** 1 Department of Automatic Control and System Engineering, Faculty of Engineering in Bilbao, University of the Basque Country (UPV/EHU), Plaza Ingeniero Torres Quevedo 1, 48013 Bilbao, Spain; aitziber.mancisidor@ehu.eus (A.M.); itziar.cabanes@ehu.eus (I.C.); eva.portillo@ehu.eus (E.P.); 2 Neurorehabilitation Area, Health Division, TECNALIA Research and Innovation, Mikeletegi Pasealekua 1-3, Donostia-San Sebastian 20009, Spain; jehyung.jung@tecnalia.com

**Keywords:** rehabilitation robotic device, Force Virtual Sensor, motion virtual sensor, advanced controller

## Abstract

In order to properly control rehabilitation robotic devices, the measurement of interaction force and motion between patient and robot is an essential part. Usually, however, this is a complex task that requires the use of accurate sensors which increase the cost and the complexity of the robotic device. In this work, we address the development of virtual sensors that can be used as an alternative of actual force and motion sensors for the Universal Haptic Pantograph (UHP) rehabilitation robot for upper limbs training. These virtual sensors estimate the force and motion at the contact point where the patient interacts with the robot using the mathematical model of the robotic device and measurement through low cost position sensors. To demonstrate the performance of the proposed virtual sensors, they have been implemented in an advanced position/force controller of the UHP rehabilitation robot and experimentally evaluated. The experimental results reveal that the controller based on the virtual sensors has similar performance to the one using direct measurement (less than 0.005 m and 1.5 N difference in mean error). Hence, the developed virtual sensors to estimate interaction force and motion can be adopted to replace actual precise but normally high-priced sensors which are fundamental components for advanced control of rehabilitation robotic devices.

## 1. Introduction

Global aging and a sedentary lifestyle in industrialized countries have increased the number of patients with reduced mobility due to stroke [1]. Nowadays, more than 33 million people around the world have to live with stroke sequels [2]. In order to recover the lost mobility and improve the quality of life of these patients, it is necessary to perform appropriate rehabilitation treatments which usually require several years to provide the desired results [3]. However, in most rehabilitation programs trainings hours are limited due to staff and financial constraints [4].

Due to the need of increasing the effectiveness and the resources of rehabilitation for stroke patients, over last couple of decades robotic devices have been proposed to execute upper [5,6] and lower limb [7,8,9] rehabilitation programs. Rehabilitation robots emulate and replicate the movements and exercises performed by a physiotherapist, obtaining treatments of higher intensity, frequency and repetitiveness that can allow better recovery of patients with reduced mobility [3]. In addition, they can work as a measurement tool of the interaction forces and motions, providing data for the analysis of the state of recovery of the patient [4]. Finally, integrating an adequate graphic interface, they can motivate the patients to continue with the rehabilitation program by using games [10].

The long training process is composed by several phases, in which the patient mobility is gradually increased [11]. Hence, robotic devices need to adapt their performance and task execution to the particular needs of each patient. This way, in the early stages of rehabilitation, patients typically cannot move autonomously, and the movement must be executed by the robot. After a few months of training, patients gradually recover part of their mobility, and they must be encouraged to generate movement on their own. This is, passive training mode is required for initial stages, while the so-called active training mode must be used in more advanced phases [12].

In order to implement these training modes, it is necessary to control both the force and motion of the robot/user interaction. In passive mode, a suitable position controller is necessary, so that the robotic device executes the desired trajectory. On the other hand, in active mode, a force controller is required to assist the user to perform the rehabilitation task [13]. These controllers calculate the torque/force to be applied by the actuators of the robotic device based on the reference generated by the selected training mode (passive or active) and the robot/user interaction feedback (force or motion).

Usually, this interaction feedback is measured directly using position and force sensors. However, although various research groups work in this line [14], there are still no specific sensors for this type of applications. Hence, most rehabilitation robots that can be found in the literature use industrial sensors, which are not adapted for training therapies [15,16,17] and present several drawbacks: (1) rehabilitation robots usually present complex structures, and the placement of sensors in the user/robot interaction point is a challenging task; (2) the introduction of a number of sensors in wearable robots may increase their weight, which is not desirable; (3) noise and temperature dependency of the force sensors require adequate processing of sensors signals; and finally; (4) the use of accurate sensors increases the cost of robotic rehabilitation devices, reducing its market growth [18].

The lack of proper sensors to measure the robot-human interaction has motivated some researchers to develop sensorless controllers. Usually, these advanced algorithms estimate the interaction forces and motions based on the dynamic/kinematic model of the robot and the measurements provided by the integrated sensors of the actuator systems [19,20]. However, most of sensorless approaches are focused on industrial [21] or surgical [22] applications, while very few are applied to rehabilitation robots [23], where the main focus is estimating the robot-human interaction.

In this context, this work presents the use of virtual sensors as an alternative to direct measurement using accurate sensors in rehabilitation robotics. Virtual sensors allow to estimate the robot/user interaction force and motion, reducing the overall cost and the complexity of the robots. For that purpose, a kinematical/dynamical mathematical model of the robotic device and low cost position sensors, such as optical encoders or linear potentiometers are used. This approach is applied to the Universal Haptic Pantograph (UHP) upper limb rehabilitation robot, used for the training of shoulder, elbow and wrist in people who suffer motor deficit after a stroke [24].

The rest of this paper is organized as follows: Section 2 introduces the Universal Haptic Pantographth rehabilitation robot. Section 3 describes the concept and theoretical development of the presented force and motion virtual sensors. The approach is validated Section 4, comparing the performance of the robot controller with real sensors and virtual ones. Finally, the most important ideas are summarized in Section 5.

## 2. UHP Rehabilitation Robot

**Figure 1:**
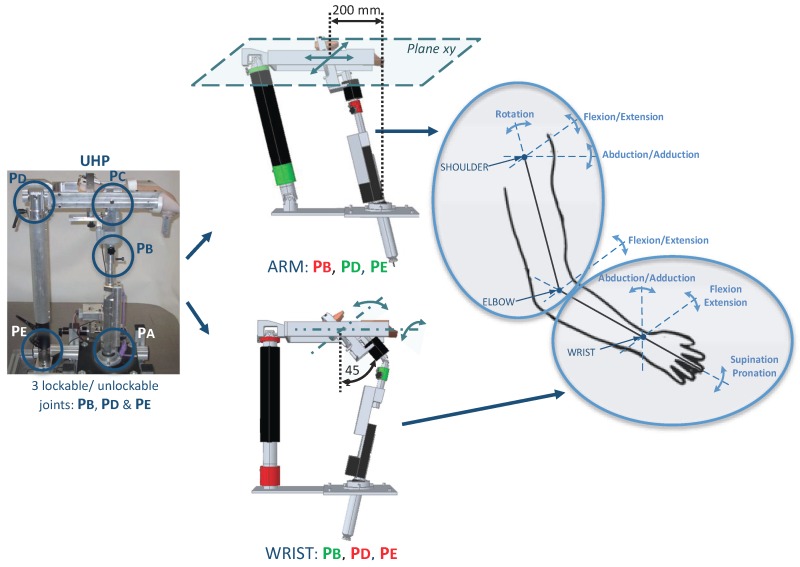
ARM and WRIST operating modes of the UHP rehabilitation robot (Red color denotes locked joint, Green color denotes unlocked joint).

The UHP robotic device is a multifunctional upper limb rehabilitation robot designed for the treatment of people with reduced mobility due to stroke [24]. Thanks to the use of three lockable/unlockable joints ($P_{B}$, $P_{D}$ and $P_{E}$), the UHP has the ability to configure its mechanical structure (Figure 1). This reconfigurable structure allows to define eight operating modes. However, for the case of study analyzed in this work, two of the most used will be detailed: ARM and WRIST modes [25].

The *ARM operating mode* allows to perform planar motions that result in arm flexion/extension exercises. This allows to execute rehabilitation exercises associated with the three movements of the shoulder (rotation, flexion/extension and abduction/adduction) and the movement of flexion/extension of the elbow [26]. On the other hand, *WRIST operating mode* allows to perform rehabilitation exercises associated with the rotating movements of the wrist (abduction/adduction and flexion/extension) and the supination/pronation movement of the forearm [27]. So, all joints of the upper limb can be trained by combining these two operating modes (Figure 1).

**Figure 2:**
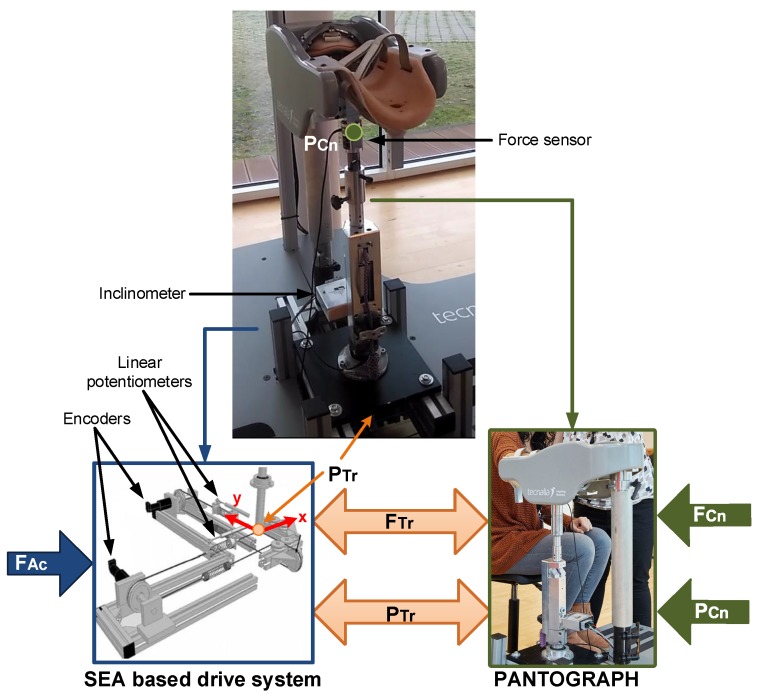
Two main subsystems of the UHP rehabilitation robot.

The UHP rehabilitation robot is divided into two subsystems: a pantograph-based robotic device and a drive system based on elastic components (Figure 2). The pantograph structure interacts with the patient at the contact point ($P_{\mathrm{Cn}}$), while the drive system provides the force/motion to the robotic device by means of two ortogonally placed Serial Elastic Actuators (SEA) [28]. Each SEA actuator is composed of a rotary motor and two linear springs connected by rigid cables, as shown in Figure 2. Hence, the motion of the drive system based on elastic components ($P_{\mathrm{Ac}}$) is generated by the rotative motions of the motors ($q_{m}$) and the variable length of the springs ($n_{S}$). The two subsystems are connected in a single transmission point ($P_{\mathrm{Tr}}$).

In order to measure the actuator system motion ($P_{\mathrm{Ac}}$), the UHP rehabilitation robot has two optical rotative encoders (HEDS-5500 of Avago technologies, 1024pulses/revolution resolution) and two linear potentiometers (TEX 0150 of NovoTechnik, 0,01 m resolution). The variable length of the springs ($n_{S}$) is measured with linear potentiometers, while the encoders are used to measure the rotation angle of the motors ($q_{m}$) (Figure 2).

## 3. Motion and Force Virtual Sensors

The aim of the virtual sensors is to provide an accurate feedback of the robot/patient interaction, this is, to provide estimations of the force ($F_{\mathrm{Cn}}$) and motion ($P_{\mathrm{Cn}}$) of the contact point between the robot and the patient. For that purpose, the proposed approach is based on the mathematical model of the UHP rehabilitation robot and the motion measurements provided by the SEA drive system of the robot ($P_{\mathrm{Ac}}$).

### 3.1. Motion Virtual Sensor of the UHP Rehabilitation Robot

The development of the Motion Virtual Sensor for the UHP rehabilitation robot is divided into two steps. First, the kinematical model of the drive system based on elastic components is analysed to determine the motion of the transmission point $P_{\mathrm{Tr}}$ ; second, the user/robot contact point motion $P_{\mathrm{Cn}}$ is estimated based on the kinematical model of the pantograph and the motion of the transmission point $P_{\mathrm{Tr}}$.

#### 3.1.1. Kinematical Analysis of the Drive System Based on Elastic Components

**Figure 3:**
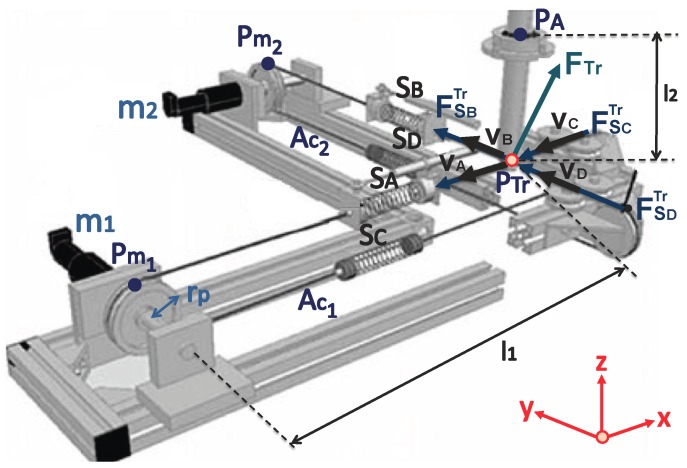
Drive system of the UHP rehabilitation robot.

As shown in Figure 2 and Figure 3, the UHP presents a single transmission point ($P_{\mathrm{Tr}}$) where 3 degrees-of-freedom motions can be transmitted bilaterally among the two subsystems.

(1) $$P_{\mathrm{Tr}}=\left[\begin{array}{c} x_{Tr}\\ y_{Tr}\\ z_{Tr} \end{array}\right]$$

The motion of the transmission point ($P_{\mathrm{Tr}}$) depends directly on the motion generated by two SEA drive systems (${\mathrm{Ac}}_{1}$ and ${\mathrm{Ac}}_{2}$),

(2) $$P_{\mathrm{Tr}}=f\left(P_{Ac_{1}},P_{Ac_{2}}\right)$$

where $P_{Ac_{1}}$ is the distance from point $P_{m_{1}}$ to the transmission point ($P_{\mathrm{Tr}}$) and $P_{Ac_{2}}$ is the distance between $P_{m_{2}}$ and $P_{\mathrm{Tr}}$ (Figure 3).

From the kinematic model of the SEA drive system [26], it is obtained by,

(3) $$\begin{array}{c} P_{Ac_{1}}=l_{1}+n_{S_{A}}-q_{m_{1}}r_{p}\\ P_{Ac_{2}}=l_{1}+n_{S_{B}}-q_{m_{2}}r_{p} \end{array}$$

where $l_{1}$ is the distance from the equilibrium point of the transmission point ($P_{0}=P_{\mathrm{Tr}}$ when $x_{Tr}=y_{Tr}=z_{Tr}=0$ m) to the motors $m_{1}$ and $m_{2}$, $n_{S_{A}}$ and $n_{S_{B}}$ are the variable lengths of springs $S_{A}$ and $S_{B}$, $q_{m_{1}}$ and $q_{m_{2}}$ are the rotation angles of the motors $m_{1}$ and $m_{2}$, and $r_{p}$ is the radius of pulleys.

On the other hand, by applying trigonometry (Figure 3), the following relationships are calculated,

(4) $$\begin{array}{c} P_{Ac_{1}}=\sqrt{{\left(x_{Tr}+l_{1}\right)}^{2}+y_{Tr}^{2}+z_{Tr}^{2}}\\ P_{Ac_{2}}=\sqrt{x_{Tr}^{2}+{\left(y_{Tr}-l_{1}\right)}^{2}+z_{Tr}^{2}}\\ x_{Tr}^{2}+y_{Tr}^{2}+{\left(l_{3}-z_{Tr}\right)}^{2}=l_{2}^{2} \end{array}$$

where $l_{2}$ is the distance from the transmission point ($P_{\mathrm{Tr}}$) to joint $P_{A}$ of the pantograph structure.

Combining Equations (3) and (4), the expression that defines the motion of the transmission point ($P_{\mathrm{Tr}}$) based on the the variables measured by the actuator sensors ($q_{m_{1}}$, $q_{m_{2}}$, $n_{S_{A}}$ and $n_{S_{B}}$) is obtained by,

(5) $$\begin{array}{c} \sqrt{{\left(x_{Tr}+l_{1}\right)}^{2}+y_{Tr}^{2}+z_{Tr}^{2}}=n_{S_{A}}-q_{m_{1}}r_{p}+l_{1}\\ \sqrt{x_{Tr}^{2}+{\left(y_{Tr}-l_{1}\right)}^{2}+z_{Tr}^{2}}=n_{S_{B}}-q_{m_{2}}r_{p}+l_{1}\\ x_{Tr}^{2}+y_{Tr}^{2}+{\left(l_{2}-z_{Tr}\right)}^{2}=l_{2}^{2} \end{array}$$

#### 3.1.2. Kinematical Analysis of the Pantograph

In order to estimate the motion of the contact point ($P_{\mathrm{Cn}}={\left[x_{Cn}\;\;y_{Cn}\;\;z_{Cn}\right]}^{T}$) based on the motion of the transmission point ($P_{\mathrm{Tr}}={\left[x_{Tr}\;\;y_{Tr}\;\;z_{Tr}\right]}^{T}$), the closure loop equation associated to the pantograph structure that relates both parameters has to be defined as,

(6) $$\Gamma (P_{\mathrm{Tr}},P_{\mathrm{Cn}})=0$$

**Figure 4:**
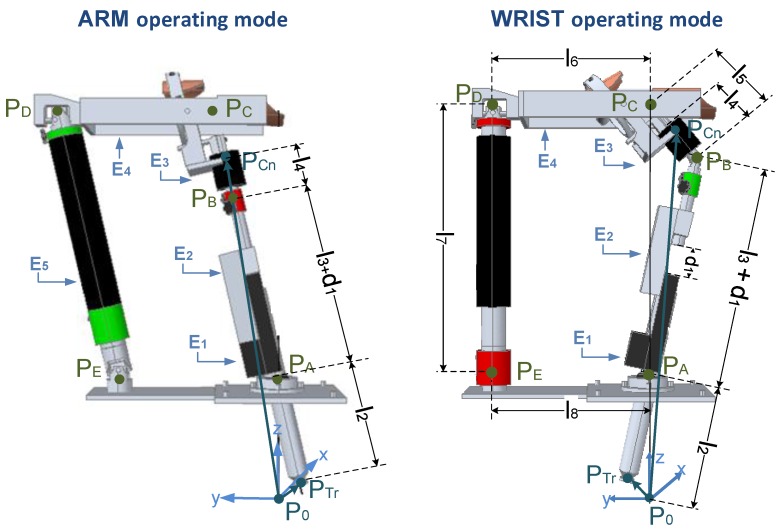
Pantograph structure of the UHP rehabilitation robot.

Equation (6) depends on the mechanical configuration of the pantograph structure, this is, on the particular operating mode (ARM or WRIST) selected (Figure 4).

**ARM operating mode**

In ARM operating mode, joint $P_{B}$ is locked. Hence, the closure loop equation is (Figure 4),

(7) $$P_{\mathrm{Tr}}+l_{2}+l_{3}+d_{1}+l_{4}-P_{\mathrm{Cn}}=0$$

solving for $P_{\mathrm{Cn}}={\left[x_{Cn}\;y_{Cn}\;z_{Cn}\right]}^{T}$,

(8) $$\left[\begin{array}{c} x_{Cn}\\ y_{Cn}\\ z_{Cn} \end{array}\right]=\left[\begin{array}{c} x_{Tr}\\ y_{Tr}\\ z_{Tr} \end{array}\right]+\frac{l_{2}+l_{3}+d_{1}+l_{4}}{l_{2}}\left[\begin{array}{c} -x_{Tr}\\ -y_{Tr}\\ l_{2}-z_{Tr} \end{array}\right]=\frac{-l_{3}-d_{1}-l_{4}}{l_{2}}\left[\begin{array}{c} x_{Tr}\\ y_{Tr}\\ z_{Tr} \end{array}\right]+\left[\begin{array}{c} 0\\ 0\\ l_{2}+l_{3}+d_{1}+l_{4} \end{array}\right]$$

**WRIST operating mode**

On the other hand, in WRIST operating mode, joint $P_{B}$ is unlocked and $P_{D}$ and $P_{E}$ are locked. Hence, the closure loop equation can be calculated as (Figure 4),

(9) $$l_{3}+d_{1}+l_{5}+l_{6}-l_{7}-l_{8}=0$$

where $l_{3}+d_{1}$ depends on the motion of the transmission point ($P_{\mathrm{Tr}}$) and $l_{6}$ depends on the motion of the contact point ($P_{\mathrm{Cn}}$),

(10) $$\frac{\left(l_{3}+d_{1}\right)}{l_{2}}\left[\begin{array}{c} -x_{Tr}\\ -y_{Tr}\\ l_{3}-z_{Tr} \end{array}\right]+\frac{l_{5}}{l_{5}-l_{4}}\left[\begin{array}{c} -x_{Cn}\\ l_{6}-l_{8}-y_{Cn}\\ l_{2}+l_{7}-z_{Cn} \end{array}\right]-\left[\begin{array}{c} 0\\ l_{8}-l_{6}\\ l_{7} \end{array}\right]=\left[\begin{array}{c} 0\\ 0\\ 0 \end{array}\right]$$

Solving for $P_{\mathrm{Cn}}={\left[x_{Cn}\;y_{Cn}\;z_{Cn}\right]}^{T}$,

(11) $$\left[\begin{array}{c} x_{Cn}\\ y_{Cn}\\ z_{Cn} \end{array}\right]=-\frac{\left(l_{3}+d_{1}\right)\left(l_{5}-l_{4}\right)}{l_{2}l_{5}}\left[\begin{array}{c} x_{Tr}\\ y_{Tr}\\ z_{Tr} \end{array}\right]+\left[\begin{array}{c} 0\\ 0\\ l_{2}+l_{7}+\frac{\left(l_{5}-l_{4}\right)\left(l_{3}+d_{1}-l_{7}\right)}{l_{5}} \end{array}\right]$$

Therefore, the Motion Virtual Sensor of the UHP rehabilitation robot is defined by Equations (5) and (8) for ARM operating mode and Equation (11) in WRIST operating mode. These allow to estimate the motion of the robot/patient contact point $P_{\mathrm{Cn}}$ in terms of the sensorized variables of the drive system ($q_{m_{1}}$, $q_{m_{2}}$, $n_{S_{A}}$ and $n_{S_{B}}$): the rotation of the actuators, using encoders, and the length of the elastic springs, using linear potentiometers.

### 3.2. Force Virtual Sensor of the UHP Rehabilitation Robot

Similarly to the derivation of the Motion Virtual Sensor, the Force Virtual Sensor of the UHP is calculated in two steps: first, the dynamical model of the elastic drive system is used to estimate forces/torques generated by the actuators in the transmission point ($F_{\mathrm{Tr}}$); second, the relationship between the forces of the transmission point ($F_{\mathrm{Tr}}$) and the forces exerted at the robot/user contact point ($F_{\mathrm{Cn}}$) are estimated based on the dynamical model of the robotic device and the motion of transmission point ($P_{\mathrm{Tr}}$).

#### 3.2.1. Dynamical Analysis of the Drive System Based on Elastic Components

As shown in Figure 3, the transmission force ($F_{\mathrm{Tr}}$) of the UHP rehabilitation robot is the sum of the forces exerted by the four springs (A,B,C,D) that are connected to the transmission point ($P_{\mathrm{Tr}}$),

(12) $$F_{\mathrm{Tr}}=F_{{\mathrm{AC}}_{1}}+F_{{\mathrm{AC}}_{2}}=F_{S_{A}}+F_{S_{B}}+F_{S_{C}}+F_{S_{D}}$$

where each spring force ($F_{S_{i}}$) is calculated using Hooke’s law based on its variable length ($n_{S_{i}}$), its stifness constant ($K_{S_{i}}$) and its unitary directional vector (${\vec{v}}_{i}$) [26],

(13) $$F_{S_{i}}=k_{S_{i}}\;n_{S_{i}}\;{\vec{v}}_{i}$$

where ${\vec{v}}_{i}$ depends on the current position of the transmission point ($P_{\mathrm{Tr}}$), which has been defined in Equation (5).

#### 3.2.2. Dynamical Analysis of the Pantograph

Once the transmission force is estimated ($F_{\mathrm{Tr}}$), the dynamical model of the pantograph is used to estimate the robot/user contact force ($F_{\mathrm{Cn}}$). Although different formulations can be applied, in this work the Lagrangian formulation is selected [29], as it allows to calculate the dynamic model of the rehabilitation robot without considering the internal forces of the mechanism, resulting in a compact dynamic model appropriate to be implemented in advanced controllers.

The development of the dynamical model using the Lagrangian formulation requires of the calculation of both kinetic (K) and potential (U) energies of the pantograph in ARM and WRIST operating modes, whose substraction defines the Lagrangian (L).

In ARM operating mode (Figure 4), the five elements of the pantograph ($E_{1}$, $E_{2}$, $E_{3}$, $E_{4}$ and $E_{5}$) move [26]. Therefore, the Lagrangian function ($L_{\mathrm{ARM}}$) can be calculated as,

(14) $$L_{\mathrm{ARM}}=K_{\mathrm{ARM}}-U_{\mathrm{ARM}}=K_{E_{1}}+K_{E_{2}}+K_{E_{3}}+K_{E_{4}}+K_{E_{5}}-\left(U_{E_{1}}+U_{E_{2}}+U_{E_{3}}+U_{E_{4}}+U_{E_{5}}\right)$$

In WRIST operating mode (Figure 4) element $E_{5}$ is locked, so its energy is be zero. In addition, element $E_{4}$ can only rotate along its axis, so its potential energy will be zero [27].

(15) $$L_{\mathrm{WRIST}}=K_{\mathrm{WRIST}}-U_{\mathrm{WRIST}}=K_{E_{1}}+K_{E_{2}}+K_{E_{3}}+K_{E_{4}}-\left(U_{E_{1}}+U_{E_{2}}+U_{E_{3}}\right)$$

Note that in both cases $K_{E_{i}}$ represents the kinetic energy and $U_{E_{i}}$ the potential energy of element $E_{i}$,

(16) $$K_{E_{i}}=\frac{1}{2}\;\omega_{{\mathrm{CM}}_{E_{i}}}^{T}\;I_{E_{i}}\;\omega_{{\mathrm{CM}}_{E_{i}}}+\frac{1}{2}\;m_{E_{i}}\;v_{{\mathrm{CM}}_{E_{i}}}^{T}\;v_{{\mathrm{CM}}_{E_{i}}}$$

(17) $$U_{E_{i}}=m_{E_{i}}\;g\;h_{CM_{E_{i}}}$$

where $I_{E_{i}}$ is the inertia of element $E_{i}$, $m_{E_{i}}$ is its mass, $h_{CM_{E_{i}}}$ the *z* coordinate of the position of its center of mass, and $v_{{\mathrm{CM}}_{E_{i}}}$ and $\omega_{{\mathrm{CM}}_{E_{i}}}$ the linear and the angular velocity of the center of mass.

Therefore, applying the Lagrangian formulation, the relationship between the contact force ($F_{\mathrm{Tr}}$) and the transmission force ($F_{\mathrm{Cn}}$) in terms of the motion of the transmission points ($P_{\mathrm{Tr}}$) and the motion of the contact point ($P_{\mathrm{Cn}}$) is obtained.

(18) $$\begin{array}{c} \frac{d}{dt}\left(\frac{\partial L}{\partial {\dot{P}}_{\mathrm{Tr}}}\right)-\frac{\partial L}{\partial P_{\mathrm{Tr}}}=\sum \lambda_{i}\frac{\partial \Gamma (P_{\mathrm{Tr}},P_{\mathrm{Cn}})}{\partial P_{\mathrm{Tr}}}+F_{\mathrm{Tr}} \end{array}$$

(19) $$\begin{array}{c} \frac{d}{dt}\left(\frac{\partial L}{\partial {\dot{P}}_{\mathrm{Cn}}}\right)-\frac{\partial L}{\partial P_{\mathrm{Cn}}}=\sum \lambda_{i}\frac{\partial \Gamma (P_{\mathrm{Tr}},P_{\mathrm{Cn}})}{\partial P_{\mathrm{Cn}}}+F_{\mathrm{Cn}} \end{array}$$

where, $\lambda_{i}$ is the set of Lagrange multipliers and $\Gamma (P_{\mathrm{Tr}},P_{\mathrm{Cn}})$ is the closure loop equation that relates $P_{\mathrm{Tr}}$ and $P_{\mathrm{Cn}}$, as defined in Section 3.1.2.

Combining this relationship with Equation (12) the Force Virtual Sensor of the UHP rehabilitation robot is obtained, which estimates robot/patient contact force $F_{\mathrm{Cn}}$ in terms of the sensorized variables of the drive system ($q_{m_{1}}$, $q_{m_{2}}$, $n_{S_{A}}$ and $n_{S_{B}}$).

## 4. Validation and Results

**Figure 5:**
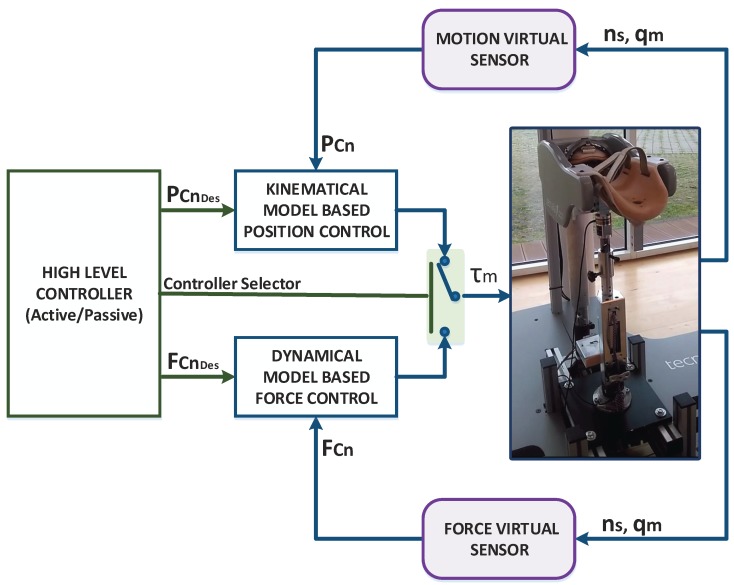
Advanced Controller for the UHP rehabilitation robot with Motion and Force Virtual Sensors.

In order to validate the developed Virtual Sensors, they were integrated into an advanced position/force controller designed for the UHP rehabilitation robot and their performance was evaluated by performing active and passive rehabilitation exercises. The integrated controller is composed by a set of controllers that operate at two levels. In the low level, two controllers operate in parallel: a kinematical model-based position control and a dynamical model-based force control. The high level controller, which defines the rehabilitation task (active or passive), is used to select the force or position controller and their reference. The overall structure of the controller is summarized in Figure 5.

As shown in Figure 5, the low level position and force controllers require contact point motion ($P_{\mathrm{Cn}}$) and contact force ($F_{\mathrm{Cn}}$) measurements respectively to operate properly. Hence, the Virtual Sensors were integrated in the control loop to estimate these variables instead of measuring them using high accuracy sensors.

**Figure 6:**
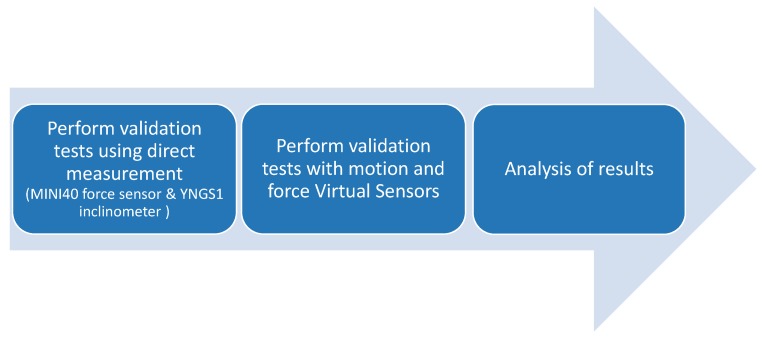
Procedure to evaluate the applicability and reliability of the developed Virtual Sensors.

To evaluate the applicability and reliability of the Virtual Sensor approach, the control performance of the robot based on the proposed Virtual Sensors has been compared with that using actual high accuracy sensors in different experimental tests. For that purpose, a three step procedure has been followed for the validation, as shown in Figure 6: (1) Each experimental test has been carried out with the rehabilitation robot, where the required low level controllers have been fed with the data provided by high accuracy sensors that measure $P_{\mathrm{Cn}}$ and $F_{\mathrm{Cn}}$; (2) the same test has been performed using controllers whose data feedback data is provided by the proposed Virtual Sensors; (3) the results of both approaches have been compared . In the first step, A MINI40 force/torque sensor (ATI, 6DOF, 1/100 N resolution) has been used to measure $F_{\mathrm{Cn}}$, while the precise YNGS1 inclinometer (Sensor-Technik Wiedemann GmbH, 3DOF, 0.25°/s resolution) has been adopted for the measurement of $P_{\mathrm{Cn}}$.

Three experimental tests have been designed to evaluate both Virtual Sensors in ARM and WRIST operating modes following the aforementioned procedure: in the first test, in order to validate the Motion Virtual Sensor, the position controller has been used, while in the last two tests, the force controller has been used to validate the Force Virtual Sensor. Results for each test are detailed next.

### 4.1. Motion Virtual Sensor Validation

The goal of the first test is to evaluate the performance of the position controller using the Motion Virtual Sensor. For this purpose, a sinusoidal reference has been designed for both *x* and *y* directional movement. The frequency varied from 0.05 Hz to 0.2 Hz as a function of time. The magnitude of the reference has been set to 0.1 m for ARM mode and 0.05 m for WRIST mode.

**Figure 7:**
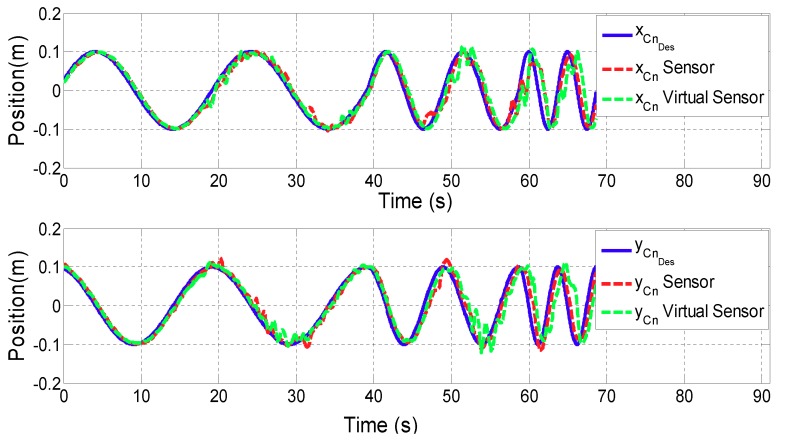
Validation of the Motion Virtual Sensor in ARM operating mode.

**Figure 8:**
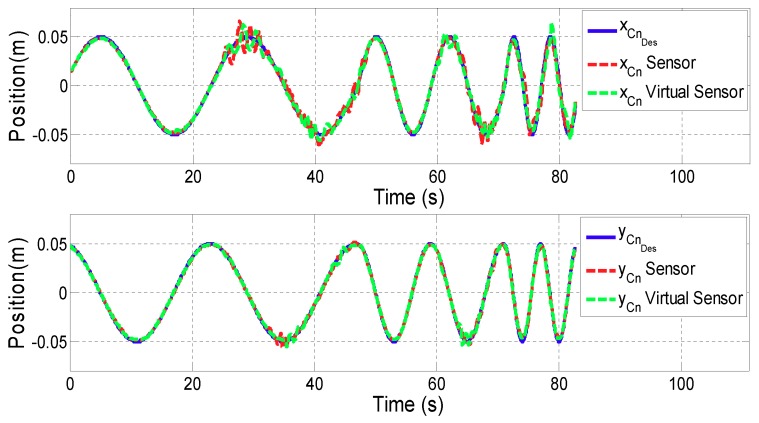
Validation of the Motion Virtual Sensor in WRIST operating mode.

Figure 7 and Figure 8 show the results of the contact point $P_{\mathrm{Cn}}$ displacement for ARM and WRIST operating modes, respectively. In the upper plot, the *x* coordinate of $P_{\mathrm{Cn}}$ is shown, while the lower one represents the *y* coordinate. In addition, the blue line illustrates the reference motion of the contact point ($P_{{\mathrm{Cn}}_{\mathrm{Des}}}$), while the red line is associated to the case using additional sensors, and the green one to the case in which the proposed Motion Virtual Sensor is used. Table 1 summarizes mean and maximum distance error $|P_{{\mathrm{Cn}}_{\mathrm{Des}}}-P_{\mathrm{Cn}}|$ for both cases.

### 4.2. Force Virtual Sensor Validation

In the following two tests, the performance of the force controller with the Virtual Sensor compared to the performance with a high accuracy sensor are analysed. The first test simulates the active mode of the robot, where the desired contact force has been set to zero ($F_{{\mathrm{Cn}}_{\mathrm{Des}}}=0\;N$). In the second one, a sinusoidal force reference with 20 N magnitude and variable frequencies of 0.05 Hz, 0.1 Hz and 0.2 Hz has been selected.

For all the figures in this section, the upper plot depicts the evolution of the *x* coordinate of the contact force ($F_{\mathrm{Cn}}$) while the lower one shows $F_{\mathrm{Cn}}$ in *y* coordinate. In addition, the blue line defines the desired contact force ($F_{{\mathrm{Cn}}_{\mathrm{Des}}}$), the red line is related to the contact force provided by the controller with real sensors ($F_{{\mathrm{Cn}}_{\mathrm{Sensor}}}$), while the green one corresponds to the case in which the Virtual Sensor ($F_{{\mathrm{Cn}}_{\mathrm{VirtualSensor}}}$) is used.

**Test 1: Zero force reference**

**Figure 9:**
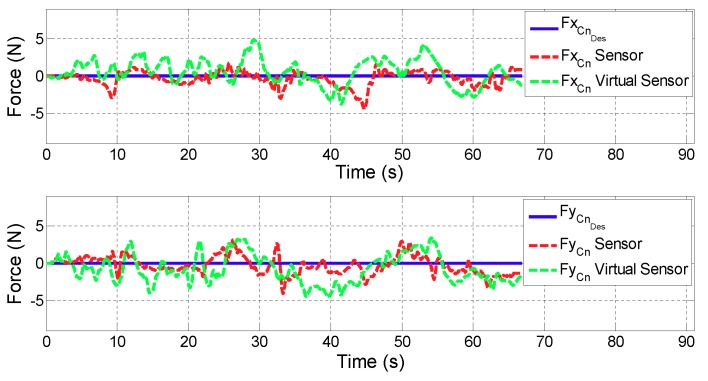
Validation of the Force Virtual Sensor in the ARM operating mode with zero force reference.

**Figure 10:**
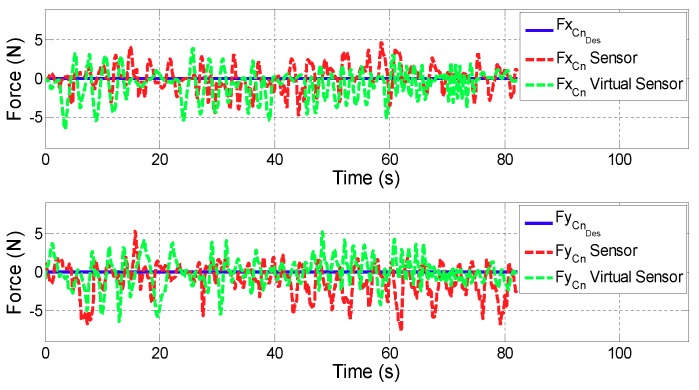
Validation of the Force Virtual Sensor in the WRIST operating mode with zero force reference.

Figure 9 and Figure 10 show the control results of zero force reference mode in ARM and WRIST operating modes. Table 2 summarizes the mean and maximum error of the force controller when the contact force is measured with the force sensor or estimated with the Force Virtual Sensor ($|F_{{\mathrm{Cn}}_{\mathrm{Des}}}-F_{\mathrm{Cn}}|$).

**Test 2: Variable force reference**

**Figure 11:**
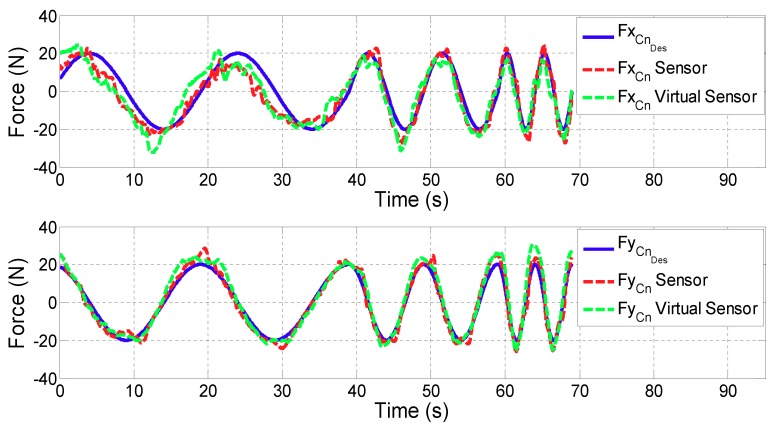
Validation of the Force Virtual Sensor in the ARM operating mode with variable force reference.

**Figure 12:**
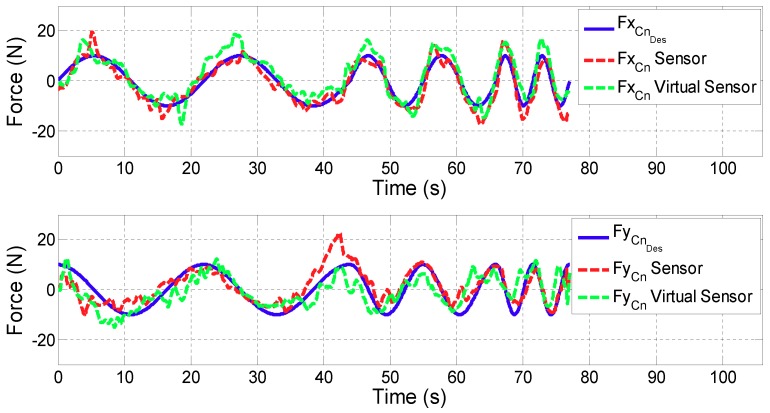
Validation of the Force Virtual Sensor in the WRIST operating mode with variable force reference.

Figure 11 and Figure 12 show the control results of variable force in ARM and WRIST operating modes, respectively. Table 3 summarizes the mean and maximum force errors carried out when considering sensor data or the proposed Virtual Sensor feedback ($|F_{{\mathrm{Cn}}_{\mathrm{Des}}}-F_{\mathrm{Cn}}|$).

### 4.3. Suitability of the Developed Virtual Sensors for Advanced Controllers in Rehabilitation Robotics

In this subsection results from the previously detailed tests will be discussed, and conclusions drawn. The results of the first validation test show that the performance of the position controller using Virtual Sensors is similar to the one with real sensors. According to the literature [30], healthy people are able to perform movements of the upper extremities with an average resolution of 0.005 m. Therefore, it can be stated that the obtained error difference (0.005 m in ARM and 0.0001 m in WRIST operating mode) between virtual and real sensor is acceptable for rehabilitation therapies.

On the other hand, results of the second validation test show that in ARM operating mode, the performance of the force controller in active rehabilitation tasks (zero force reference) is very similar when considering actual sensor data, or the proposed Virtual Force Sensor. The existing estimations errors are acceptable for rehabilitation purposes (Δ of force error <0.6 N) due to the sensitivity limit of humans. The performance of the controller when using Virtual Sensors is even better than the use of real sensor data in WRIST mode due to the mechanical configuration of the robot and its influence on the force sensor (Δ of the mean error =−0.07 N and Δ of the maximum error =−1.15 N). It is also important to note that the tests have been carried by a healthy person, which has moved arbitrarily and randomly the robot in this case. This way, the misaligment of peaks in Figure 9 and Figure 10 are due to being different executions of the same test.

In the proposed third validation test, in which variable force references are tested, the controller has been able to follow the desired force reference with small and acceptable errors for both cases. The difference between the two implementation (sensor and Virtual Sensor) is almost negligible for the user (Δ of the average error is 1.5 N in ARM and 0.85 N in WRIST operating mode). In addition, it should be noted that these tests have been carried out by a healthy person, who can exert much more force than a stroke patient. Hence, less error is expected for these cases.

It is important to emphasize that in a rehabilitation application, safety and robustness of the device are more critical than the precision of the movements [31,32]. For these applications, fast controllers capable of responding quickly to the movement generated by the patient are required. Therefore, the use of compact Virtual Sensors like ones proposed are preferred to complex and precise Virtual Sensors that need more computational cost and slow down the speed of the controller.

Therefore, after performing different validation tests with the UHP rehabilitation robot in ARM and WRIST operating modes, it is concluded that the developed Force and Motion Virtual Sensors are suitable for their use in advanced controllers for rehabilitation robots.

## 5. Conclusions

Robotic devices can improve the rehabilitation process of patients with reduced mobility after a neurological disorder, such as a stroke. One of the key components for successful robot-mediated therapy is to design advanced controllers that can adjust interactive force and motion between the patient and the robot as needed. Force and motion values are usually measured through accurate sensors, which increases the cost and complexity of the device.

In this work, a novel virtual sensor approach is proposed for the estimation of the contact force and motion in rehabilitation robots. The developed Virtual Sensors are based on the mathematical model of the robotic device and the measurement provided by low cost position sensors, such as optical encoders or linear potentiometers.

The proposed Virtual Sensors have been implemented and evaluated in an advanced force/position controller of the UHP rehabilitation robot for upper limb training. Control performance based on Virtual Sensors has been compared to that using real sensors in performing passive and active rehabilitation exercises with a healthy subject through three different experimental conditions.

Results shows that the use of the Virtual Sensors produces performance similar to that obtained with real sensors in terms of motion and force errors (less than 0.005 m of mean motion error and 1.5 N of mean force error), which is acceptable for rehabilitation use. While the results reveal that Virtual Sensors can be used as an alternative of real sensors, further experiments with actual patients who have different level of impairments will be future interesting topic in order to ensure clinical adaptation and acceptance of the developed Virtual Sensors.

## Figures and Tables

**Table 1:** Mean and maximum motion errors of the position controller.

Operating	With the Sensor		With the Virtual Sensor		Δ of the Error	
Mode	*Mean*	*Maximum*	*Mean*	*Maximum*	*Mean*	*Maximum*
ARM	0.015 m	0.098 m	0.02 m	0.141 m	0.005 m	0.043 m
WRIST	0.0022 m	0.021 m	0.0021 m	0.015 m	0.0001 m	0.007 m

**Table 2:** Mean and maximum errors of the force controller with zero force reference.

Operating	With the Sensor		With the Virtual Sensor		Δ of the Error	
Mode	*Mean*	*Maximum*	*Mean*	*Maximum*	*Mean*	*Maximum*
ARM	0.88 N	4.38 N	1.42 N	4.87 N	0.54 N	0.49 N
WRIST	1.45 N	7.72 N	1.38 N	6.57 N	−0.07 N	−1.15 N

**Table 3:** Mean and maximum errors of the force controller with variable force reference.

Operating	With the Sensor		With the Virtual Sensor		Δ of the Error	
Mode	*Mean*	*Maximum*	*Mean*	*Maximum*	*Mean*	*Maximum*
ARM	2.89 N	14.72 N	4.34 N	14.61 N	1.45 N	−0.11 N
WRIST	2.92 N	14.10 N	3.77 N	15.20 N	0.85 N	1.10 N

## Abbreviations

The following abbreviations are used in this manuscript:

SEA	Serial Elastic Actuator
UHP	Universal Haptic Pantograph
